# Compound Fractures1Read before the Central New York Medical Association, November 18, 1911, and he Erie County Medical Society, April 22, 1912.

**Published:** 1912-08

**Authors:** Thew Wright

**Affiliations:** Attending Surgeon Emergency Hospital; Assistant Surgeon Buffalo General, Erie County, and Children’s Hospitals Buffalo. N. Y.


BUFFALO MEDICAL JOURNAL

VOL. LXVIII. AUGUST, 1912. No. 1

### Compound Fractures[1]

By THEW WRIGHT, M.D.
Attending Surgeon Emergency Hospital
Assistant Surgeon Buffalo General, Erie County, and Children's Hospitals
Buffalo, N. Y.

Instead of taking up your time in the consideration of the popular surgical topics of the day, I am going to ask your attention for a few moments upon a subject as old as the art of surgery, as interesting as you choose to make it, and as important as any with which we are called upon to deal. I know of no branch of surgery in the practice of which versatility, ingenuity, conservatism, quick decision and good judgment are more essential to the securing of good results than in the treatment of compound fractures. To be sure these injuries have lost much of the dread with which they were viewed before the introduction of Lister's methods, when the sugeon's choice lay between primary amputation and death from infection, and when primary amputation often failing to save life the mortality from compound fractures was between 40 and 50%. With the introduction of antiseptic methods the mortality decreased to less than 10%. But these injuries still remain, perhaps, the most important division of traumatic surgery.

Compound fractures are defined in many ways. A satisfactory definition is that a compound fracture is one in which there is a more or less direct communication between the seat of fracture and the external air through a wound in the skin and soft parts. This communication may be brought about by the force which causes the fracture lacerating the soft parts on its way to reach the bone, or one of the fragments of the bone may produce the laceration. A fracture may be simple at first and become compound secondly, either by faulty handling allowing jagged bone ends to pierce the skin or by anything which will cause a dissolution of the continuity of the soft parts overlying the fracture, as for example the infection of a blister.

Every step in the treatment of these injuries is of importance and in order that our discussion may follow in logical sequence

1. Read before the Central New York Medical Association, November 18, 1911, and he Erie County Medical Society, April 22, 1912.

let us consider for a moment the question of "first aid." That is, what shall we do and what shall we not do if called to such a patient on the street in the factory or on the road. The clothing should be cut open with a knife or shears so that it may be readily removed without being rubbed and dragged across the site of injury, thereby adding to the probability of infection. A compound fracture may be considered as an infected wound in practically every instance. It was this fact of the presence of infection of course which made these injuries so formidable in the preantiseptic era. But care must be taken not to add to the infection already present. After the wound is exposed it should be covered with sterile gauze, if such is at hand, or if that is lacking, with the cleanest available cloth, which should be snugly bandaged in place. Should severe hemorrhage persist in spite of this, it may be necessary to apply a tourniquet.

The extremity, if it be one, should now be immobilized in a pillow, blanket or board splint, usually without attempt to correct the deformity which may be present and always without such attempt if a fragment of bone is protruding from the wound. The reduction of such a fragment before it is thoroughly cleaned may readily cause the introduction of infectious material into an otherwise comparatively clean wound. There should be no handling of the wound at this time and the immobilization should be so complete that there is the least possible disturbance to the injured part during transportation to the patient's house, the doctor's office or, what is always the best place for such injuries, the hospital.

If the fracture be of an extremity, the first question that arises is, can the limb be saved or will it require amputation? This is a question that often cannot be answered at this time. I have seen limbs apparently hopelessly crushed which were saved and ultimately restored to usefulness. Unless the force which caused the fracture has cut off all the principal arteries supplying the part, I believe a primary amputation is never justifiable and that in the vast majority of cases, with proper treatment, the part will be saved. I have never primarily amputated an extremity in which there was any demonstrable arterial circulation and altho a subsequent amputation has been required in some cases, there have been enough instances in which this was not the case, that I feel that we cannot easily be too conservative in the matter. If the points upon which I shall touch are carefully carried out we have very little to lose by conservatism and usually much to gain.

To be sure there are cases of compound comminuted fractures, especially of the foot and ankle, where we can be sure that even though the part be saved the best possible outcome, even after months of treatment, would leave a member so distorted and deformed as to be no better than an artificial limb. In such a case primary amputation should be done. This will be more often justifiable in the leg than in the forearm, for the loss of the hand is a far more serious matter than that of a foot in these days of excellent, prosthetic work and it is justifiable to run great risks in the effort to save it. Having decided against amputation the next step is to render the injured part as nearly sterile as possible. This to my mind always means the administration of a general anæsthetic, unless we are dealing with a fracture of a phalanx, in which case local anæsthesia either by immersing the part in warm carbolic solution or by injecting cocaine will often suffice.

The thorough cleansing and primary dressing of a compound fracture of a large bone can only be properly carried out with the patient asleep. Ether, always the safest anæsthetic, is especially indicated in this class of cases where preliminary preparation is impossible.

As to our method of attempting to sterilize the injured parts I feel we can not be too dogmatic in our statements. I feel that here, as in other fields of surgery, we must suit our methods to the individual case at hand. The man who says he always does this or always does that will ofttimes err, not in judgment for the very fact that he always follows a routine precludes the use of judgment. Right here in the choice of methods of sterilizing the wound we are called upon to exercise most careful judgment.

There are men who say, always scrub the skin thoroughly with soap and water, then scrub the wound in the same manner. Others say, never scrub either one or the other, but rely upon our best antiseptic iodine to sterilize.

In deciding the method to be used we must take into consideration the way in which the compound element of the fracture was produced as well as the extent of the laceration of soft parts. If the lesion to the soft parts was produced from without inward we have certain infection of the wound, the extent of which varies with the amount of foreign matter carried into it. If the wound was made by the protrusion of the bone end we have probably no infection in the wound and if we can thoroughly disinfect the surrounding skin, and the bone end before replacing it we will have none at any time. In this latter class of cases the best results will be obtained by cleansing the surrounding skin with gasoline and then applying iodine, treating the protruding bone end in the same manner. If we find dirt ground into the bone end, and this cannot be easily removed, it will be wise to clip or rongeur away the soiled surface and then apply the iodine to it. The fragment may then be replaced, a sterilized gauze dressing applied and in the vast majority of cases healing will be prompt. It must be admitted that the body can take care of a considerable amount of infection and that in the majority of cases the local reaction will overcome infection unless it be of an especially virulent type. None the less, in my opinion we are always running a risk if we suture or primarily close the skin over a compound fracture, and it will be far safer if we insert a small strip of oil silk or gutta percha as a drain.

Instead, however, of merely replacing the fragment, in many cases it will be wiser to enlarge the wound by incision, otherwise comminution of bone and accumulation of clot sufficient to cause trouble may be overlooked.

In the more extensive lacerated wounds produced from without inward it was formerly my custom always to carry out the following procedure: To cover the wound with a piece of sterile gauze in order to prevent washing the dirt from the surrounding skin into it, then to scrub the whole extremity thoroughly with green soap, water and brush and rinse it off with sterile water. To treat the wound in the same manner, to wash the limb including the wound with ether, thoroughly cleanse the wound with turpentine, swab it out with tincture of iodine and finally wash it out with alcohol.

Turpentine is an excellent medium for removing grease and grime from wounds and is also a good antiseptic. That this method of treating these wounds is efficient has been amply demonstrated by many surgeons. But as our knowledge of the possibilities of iodine has increased we have learned to rely more and more upon it. It is now the exception for me to scrub and irrigate such a wound. We have found that by far the best results are obtained in most accidental wounds by omitting all scrubbing and irrigation, which a few years ago were thought to be essential, and relying upon tincture of iodine to accomplish the sterilization of the wound, using forceps to remove foreign bodies and cutting away with scissors or knife all shreds of severely mutilated and evidently infected tissue.

Iodine properly applied accomplishes several things. First, there is no other antiseptic so slightly toxic which covers so wide a variety of pathogenic bacteria; second, it sears over the wounded surface, closing the ends of the small cut vessels and lymphatics and thus greatly limits the spread of infection. It is often impossible by any means at our command to render these wounds perfectly sterile, but tincture of iodine by inhibiting the growth of bacteria and by its action on the tissues prevents the spread of infection from such small areas as may not have been rendered so.

In using tincture of iodine for skin disinfection the point has been made and is generally accepted that the skin must be absolutely dry. This is because water causes the epithelium to swell and thereby prevents the deeper penetration of the drug. This objection does not apply to the case of irrigation of the deeper tissues for by the very nature of things we are dealing with wet structures—I therefore feel there are occasional cases where the method outlined will give better results than when washing is omitted. That is, cases where we have a large amount of foreign material carried into the wound. But as a routine measure I think it should be discarded. In order to get at pockets containing dirt it is often necessary to enlarge the wound in various directions and to trim lacerated skin edges and even cut off portions of muscle and fascia.

After thoroughly disinfecting the wound it should be carefully inspected to determine the exact condition of the fracture. This should be done with as little handling as possible and the surgeon should wear gloves. Many wounds have been first made septic by the careless handling of the over-enthusiastic surgeon. Torn vessels should be ligated, and tendons, nerves, pieces of fascia, or muscle should be carefully removed from between the bone ends. Loose fragments of bone, i. e., those detached from their periosteum, should be removed. We must now decide how much to do in the way of repairing torn nerves, tendons, muscles, and fascia, as well as how to drain, (for I believe we should always drain.) Regarding the first point I would say that we should be governed by the amount of laceration and evident infection. If the tissues are badly crushed and dirt and grime have entered the wound in considerable quantity the less plastic work attempted the better for in the presence of infection it will fail and much plastic work may materially increase the primary shock. Any unnecessary manipulation of such wounds increases the danger of sepsis and may add just enough to the insult already done the tissues to turn the tide against recovery.

In such cases our entire effort should be directed toward saving the limb and getting as good bony apposition as possible. I feel sure that efforts to do too much at the primary dressing have frequently been the cause of secondary amputations. Plastic work can well be deferred until a later date when the wound has been healed sometime and the tissues have regained their vitality. Above all things there should be no attempt to better bony apposition by the introduction of foreign bodies, such as silver wire, bone plates or splints. In the first place good apposition can as a rule be obtained without such means. But even if this is not possible it is far wiser to wait until the wound has healed and then perform whatever operation may be necessary.

The manipulations which necessarily accompany the introduction of bone plates, wires, etc., as a primary procedure will often make the wound septic if it is not already so. They will often add such additional trauma to the partly devitalized tissues and so further damage the circulation as to necessitate amputation of a part that would otherwise have been saved. Furthermore bone plates and wire when introduced as a primary procedure, even when they do not produce such dire results as these, rarely accomplish the purpose for which they are used. They practically always act as irritants and cause more or less bone necrosis, are foreign bodies in the strictest sense of the word and preventing healing so long as present, must eventually be removed. During the past few years since Dr. Lane's procedure of operating upon simple fractures and the use of his bone plates have been received with increasing favor, there have been many instances in which, forgetting the principles he lays down for the application of his methods, surgeons have introduced these plates as a primary procedure. The first fundamental and by all means most important element in the successful use of bone plates or splints perfect asepsis, is practically never present in these cases. To the surgeon of experience these facts are familiar and mentioning them may seem superfluous, but I have so many times had to explain to the family doctor why I did not plate or wire in a given case that I presume on your good nature by calling attention to them. We must remember that our first efforts are to save the extremity in cases of severe injury and must ever be on guard against the temptation to do too much. How little it profits the patient to have a beautiful example of the joiners' art in a limb that he will eventually lose. Furthermore we must not be too ready to perform secondary operation. We should strive for good functional results rather than for a perfect restoration of bony outline as shown by X-ray, a thing rarely attainable and still more rarely necessary.

How much needless work the X-ray has caused it would be hard to estimate, but let us not be led too easily to attempt to better slight displacements demonstrated by it.

Now as to the question of drainage. I believe we should always drain these wounds. That is, we should allow free exit for the serum and blood that collects. We know that it is only when pus and the toxins it contains are under tension that they cause appreciable harm. If we allow these wounds to be closed at once by clotting or skin-edge adhesion we seal a container of excellent culture medium and we produce the most ideal condition for the absorption of any toxins formed. With proper drainage we prevent the accumulation of this pabulum for bacterial growth, we allow the lymph stream to pour out serum which shall carry infection away and we prevent the absorption of toxins. It is rare to see any alarming toxemia from a wound that is draining freely. Some surgeons advocate no drainage until the signs of sepsis appear but this seems to me to savor too much of locking the barn door after the horse is stolen, and I feel that drainage plays an important part in prophylaxis in these cases. For material oil silk, gutta percha tissue, raffia, or occasionally the fenestrated rubber tubes are the methods of choice. Plain gauze only acts as a plug and soon defeats its purpose.

The next question is regarding the means of securing proper immobilization. This is made more difficult in this type of fracture by the necessity of so arranging our retention apparatus that the wound may be dressed without unduly disturbing the limb. As a rule I think this purpose is best accomplished by a fenestrated plaster cast. The wound is covered with a sterile gauze dressing, held in place by a gauze bandage. The limb is then wrapped in sheet wadding and a circular cast applied. After the plaster is set but while still soft, it should be split lengthwise to allow for spreading, if subsequent swelling of the limb occurs of sufficient extent to call for it. A window sufficiently large to permit of easily dressing the wound is then cut in the cast. In order that the removal of an area sufficiently large to allow easy dressing may not weaken the cast it will often be necessary to incorporate strips of metal or wood when applying the plaster. There are several ways of protecting the edges of the fenestrum from becoming soiled by the discharge from the wound, the simplest and best way I have found to be that suggested by Dr. Crouse, of El Paso, Texas. Sufficient dental rubber is dissolved in chloroform to make a thick mucilagenous paste, into this small pieces of lamb's wool are cut and thoroughly mixed. After cleansing the skin with ether the wool impregnated with rubber is tucked in between the edges of the cast and the skin. This makes an impervious and elastic dam which effectually walls off the wound area and allows the cast to remain sweet and clean in spite of profuse discharge from the wound.

Finally as a routine in every case of compound fracture antitetanic serum should be injected within a few hours after the injury. Without this prophylactic measure compound fractures vie with Fourth of July injuries in the percentage of cases developing tetanus. Following its prompt use I know of no case in which the disease has developed.

Gentlemen, I thank you for this opportunity to address you upon this subject. It is one of great interest to me and one which has shared equally with any in the advances made in surgery during the past 40 years.

